# Overexpression of IGF2BP3 as a Potential Oncogene in Ovarian Clear Cell Carcinoma

**DOI:** 10.3389/fonc.2019.01570

**Published:** 2020-01-30

**Authors:** Huidi Liu, Zheng Zeng, Mitra Afsharpad, Caiji Lin, Siwen Wang, Hao Yang, Shuhong Liu, Linda E. Kelemen, Wenwen Xu, Wenqing Ma, Qian Xiang, Emilio Mastriani, Pengfei Wang, Jiali Wang, Shu-Lin Liu, Randal N. Johnston, Martin Köbel

**Affiliations:** ^1^Genomics Research Center (State-Province Key Laboratories of Biomedicine-Pharmaceutics of China), College of Pharmacy, Harbin Medical University, Harbin, China; ^2^HMU-UCCSM Centre for Infection and Genomics, Harbin, China; ^3^Translational Medicine Research and Cooperation Center of Northern China, Heilongjiang Academy of Medical Sciences, Harbin, China; ^4^Department of Biochemistry and Molecular Biology, University of Calgary, Calgary, AB, Canada; ^5^Arnie Charbonneau Cancer Institute, University of Calgary, Calgary, AB, Canada; ^6^Pathology and Laboratory Medicine, Calgary Laboratory Service, University of Calgary, Calgary, AB, Canada; ^7^Department of Pathology, Harbin Chest Hospital, Harbin, China; ^8^Hollings Cancer Center and Department of Public Health Sciences, Medical University of South Carolina, Charleston, SC, United States; ^9^Department of Microbiology, Immunology and Infectious Diseases, University of Calgary, Calgary, AB, Canada

**Keywords:** ovarian cancer, clear cell carcinoma, IGF2BP3, IMP3, prognosis, metastasis, MMPs

## Abstract

Ovarian Clear Cell Carcinoma (OCCC) displays distinctive clinical and molecular characteristics and confers the worst prognosis among all ovarian carcinoma histotypes when diagnosed at advanced stage, because of the lack of effective therapy. IGF2BP3 is an RNA binding protein that modulates gene expression by post-transcriptional action. In this study, we investigated the roles of IGF2BP3 in the progression of OCCC. We used 328 OCCCs from the AOVT (the Alberta Ovarian Tumor Type study) and the COEUR (the Canadian Ovarian Experimental Unified Resource) cohorts to elucidate the associations between IGF2BP3 expression and clinicopathological parameters, with positive IGF2BP3 expression defined as diffuse block staining, being more frequently observed at stage III (*P* = 0.0056) and significantly associated with unfavorable overall survival (HR = 1.59, 95% CI 1.09–2.33) in multivariate analysis. *IGF2BP3* mRNA gene expression was markedly increased in OCCC cell lines compared to normal tissues such as ovarian surface epithelium. We chose two IGF2BP3-overexpressing cell lines ES2 and OVMANA for *in vitro* and *in vivo* knockdown experiments. The proliferation and viability of both cell lines were significantly inhibited by two IGF2BP3 siRNAs and similar suppression was observed in cell migration and invasion by Wound Healing and Transwell assays. The percentage of apoptotic cancer cells was enhanced by both IGF2BP3 siRNAs. *In vivo* experiments showed significantly reduced sizes of tumors when treated with IGF2BP3 siRNA compared to controls. Furthermore, cancer metastasis-indicators MMP2 and MMP9 proteins were down-regulated. In conclusion, our study shows that IGF2BP3 expression is a promising biomarker for prognostication of women diagnosed with OCCC with multiple effects on key cell functions, supporting its role as an important cellular regulator with potential oncogenic activity, and as a potential target for future intervention strategies.

## Introduction

Ovarian clear cell carcinoma (OCCC) accounts for a higher proportion (up to 25%) of ovarian carcinomas among Asian than Caucasian (~10%) ethnic groups (1, 2). OCCC shows distinct morphologic features from other histotypes (i.e., high-grade serous, endometrioid, or mucinous carcinomas), that are tubulocystic, papillary, and solid architecture and glycogen-containing clear cells sometimes appearing as so-called hobnail cells. OCCCs are closely associated with ovarian and pelvic endometriosis from which they are thought to arise (3). Recurrence is not infrequent after surgery and platinum-based treatment and occurs in ~30% of OCCC patients diagnosed at stage I or II (2, 4). Therefore, better outcome prediction for stage I/II CCC patients is urgently needed. When OCCC develops to stage III or IV, the outcome is worse than high-grade serous carcinomas, because response rates to second-line chemotherapies for platinum-resistant ovarian CCCs are dismal (5). The poor prognostic outcomes with high mortality of OCCCs are largely ascribed to a lack of effective therapy. Recently, genetic alterations driving OCCC pathogenesis, such as mutations in *ARID1A, PI3CA, KRAS*, and *TERT* promoters, have been identified (6). These genetic traits may become useful markers for clinical applications, such as in early diagnosis and targeted therapy.

In our previous study of OCCC, we found that insulin-like growth factor-II mRNA-binding protein 3 (IGF2BP3 or referred to as IMP3) could be used as a biomarker to predict unfavorable prognosis in OCCC (7), which was fully supported by our later work with 73 cases from China (8). Another large study reported diagnostic utility of IFG2BP3 for OCCC (9). IGF2BP3 is a member of an evolutionarily conserved protein family involved in mRNA transport, translation and turnover by targeting the coding regions of the mRNAs (10), such as IGF2, MYC, β-catenin, β-actin, or let-7 microRNAs (11–16). IGF2BP3 has been reported to be involved in the progression of various cancers, including those in the pancreas (11), colon or rectum (17), lungs (18), and ovaries (19). According to a study with microarray assays of 8,877 human cancers and normal tissues, IGF2BP3 is associated with aggressive tumor features and unfavorable outcomes (20). In lung adenocarcinoma, overexpression of IGF2BP3 may induce the proliferation of cancer cells by mRNA degradation (21). In triple-negative breast carcinomas (TNBCs), IGF2BP3 is also associated with tumor aggression and poor outcome by participating in the EGFR-mediated migration process (22) and promotes chemoresistance by stabilizing the mRNA of ABCG2 protein (23).

Recently, studies of IGF2BP3 are rapidly increasing, mostly with a focus on its involvement in the pathogenesis of a broad range of cancers. For example, IGF2BP3 as a glioblastoma-specific marker activates the PI3K/MAPK pathways by modulating IGF-2 (24). IGF2BP3 is also involved in glioma cell migration (25). In pancreatic cancers, IGF2BP3 promotes the invasiveness and metastasis of the cancer cells through locally translated IGF2BP3-bound transcripts (26). IGF2BP3 has been reported as a potential oncogene in gastric and lung cancers via targeting p53 and miR-34a, respectively, (18, 27). In addition, IGF2BP3 is also associated with chemo-resistance and poor disease outcomes not only in OCCCs but also in other types of ovarian carcinoma, demonstrating the potential usefulness of IGF2BP3 as a novel biomarker for various tumor types, due largely to its involvement in cell invasion and metastasis. However, IGF2BP3 is still not used as a clinical biomarker.

In this study, we analyzed 328 OCCC cases using a more sensitive immunohistochemical assay to evaluate IGF2BP3 for its usefulness as biomarker for prognostication of OCCC and the reproducibility of a clinically applicable refined scoring system. We performed a series of *in vitro* and *in vivo* experiments to elucidate potential functions of IGF2BP3 in OCCC.

## Materials and Methods

### Patients and Evaluation Standards

Previously constructed tissue microarrays representing 328 OCCC cases from the Canadian Ovarian Experimental Unified Resource (COEUR, *N* = 166) and the Alberta Ovarian Tumor Type study (AOVT, *N* = 162) were used for immunohistochemistry examinations; both cohorts, described previously (28, 29), underwent full gynecopathological reviews with the integration of diagnostic biomarkers including WT1 and Napsin A1 to confirm OCCC histotypes (30). Duplicate cases were removed from analysis. Neoadjuvant chemotherapy was only occasionally administered (2%), hence, almost all samples were chemonaive. The clinicopathological features information has been detailed in Supplementary File 1.

### Immunohistochemistry Characterization

Immunohistochemistry assays were performed by Calgary Laboratory Services, Canada, and Department of Pathology, Harbin Chest Hospital, China, with tissue microarrays (TMA) on 4 μm-thick slides for each clinical sample using DAKO Omins (DAKO) and Autostainer Link 48 (Agilent, U.S.), respectively. After deparaffinization and rehydration with xylene, a series of concentrations of ethanol was used to block the endogenous peroxidase activity. For the TMA assays, we used monoclonal mouse anti-human IGF2BP3 clone 69.1 (DAKO) as primary antibody at 1:100 dilution (TRS high, 30-X-30) on a DAKO Omnis platform, with normal liver as negative control, germinal center as low expresser control and normal placenta as high expresser control (Supplementary Figure 1). Then, a 50 μL aliquot of 1:100 diluted MMP2, MMP9, or PCNA primary antibody was added to each sample. The target proteins were visualized under a microscope as brown DAB stains. IGF2BP3 staining on TMA was scored by two pathologists (M. K., M. A.) and H. D. L, who were all blinded to the clinical outcomes. Discordant cases were resolved at a multiheaded microscope. The following scoring patterns were recorded: absent, no staining; focal, staining in <10% of tumor cells; patchy/heterogeneous, staining between 10 and 90% of tumor cells; and block staining, strong diffuse staining in more than 90% of tumor cells. Only block staining was considered positive, staining less than block staining considered negative. From 41 cases, corresponding full sections were evaluated to assess the concordance between tissue microarray and full section staining.

### Cell Lines

Human ovarian OCCC cell lines (ES2, OVMANA, TOV21G, and OVTOKO) were shipped from University of Toronto, Canada, and Procell, Wuhan, China. Cells were cultured in Media 199:105, PRIM1640 or McCoy's 5A medium containing 10% FBS and penicillin-streptomycin. Cells were grown in the media at 37°C in a humidified incubator containing 5% CO_2_ in air, and passaged on alternate days with 0.05% trypsin.

### Bioinformatics Analysis

Different types of OCCC cell lines were selected for bioinformatics analysis. Simultaneously, six normal ovarian surface epithelial cell preparations (NOSEs) were used as controls to reveal the expression status of IGF2BP3. For bioinformatics analysis, we first normalized the data from the NCBI-GEO database using the six NOSEs and 10 OCCC cell lines for volcanogram and heat map analyses (GSE16570, https://www.ncbi.nlm.nih.gov/geo/query/acc.cgi?acc=GSE16570). RAmiGO gene enrichment analysis was used to detect the biological activities of IGF2BP3 in OCCC. We also applied GeneAnswers to analyze the interactions between genes among the network of IGF2BP3.

### ELISA Assay

Enzyme-linked immunosorbent assays (ELISA) were used to quantify MMP-2 and MMP-9 in cell culture supernatant. Samples or standards were dropped, 100 μL/well, into 96-well plates pre-coated with MMP2 or MMP9 antibodies, and the plates were incubated at 37°C for 1 h. After washing and drying the plate, we added HRP-conjugated secondary antibody into each well and continued the incubation at 37°C for 1 h. Substrate reagent was added, 90 μL/well, and the plates were incubated at 37°C for 15 min. Then the reaction was terminated by the addition of 50 μl/well stopping buffer. Optical intensity was determined at 450 nm immediately.

### FCM Assay

Flow cytometry (FCM) assay was used to measure cell apoptosis after treatments of the cells by NC (negative controls), siIGF2BP3-1, or siIGF3BP3-2 and washing with PBS. Then binding buffer was added to make 1 × 10^6^ cells/mL and a 100 μL aliquot of the cell suspension was moved to each flow tube. Five microliter Annexin V-FITC and 5 μL PI were dropped into tubes (NC, siIGF2BP3-1, siIGF3BP3-2). The other tubes were split into 5 μL PI, Annexin V-FITC, and double-blank for NC, siIGF2BP3-1, and siIGF3BP3-2, respectively. Each tube was wrapped in tinfoil and left at room temperature for 15 min. Another 400 μL binding buffer was added to each tube at the end of the procedure. The samples were subjected to analysis by flow cytometry (BD FACSCanto^TM^ II). The early apoptosis was evaluated based on the percentage of Annexin V positive and PI negative cells (Q4), while the late apoptosis was evaluated based on the percentage of Annexin V positive and PI positive cells (Q2). Total apoptotic cell percentages were calculated as Q2+Q4.

### Transwell Assay

Cells were washed, trypsinized and seeded in the upper chamber of transwells in serum-free medium at a density of 1 × 10^6^ cells/mL (2 × 10^5^ cells/well) on Matrigel-coated membranes, followed by the addition of NC, siIGF2BP3-1 or siIGF2BP3-2 to treat cells. All cells were grown for 48 h at 37°C for them to migrate to the lower chamber in response to medium supplemented with 10% serum in the 24-well plate. The cells on the upper layer of the membrane were removed with a cotton swab, and the membrane was fixed for 5 min with methanol. Cells on the lower side of the membrane were stained with 0.1% crystal violet and photographed by Primo Star, Carl Zeiss, Germany. Cell numbers were counted by Image-Pro Plus 6.0 software in five randomly selected fields at 100-fold magnification.

### Western Blot Analysis

ES2 or OVMANA cells were plated into three 6-well plates at 6 × 10^5^ cells/well. Each 6-well plate was divided into three groups (NC, siIGF2BP3-1, siIGF2BP3-2) and cells of each group were transfected with one of three siRNAs by Lipofectamine2000 and Opti-MEM I Reduced Serum Medium according to the manufacturer's specifications. After 48 h, cells were washed twice with PBS, lysed on ice in RIPA buffer, and then scraped off and centrifuged (12,000 r, 5 min) at 4°C. The total proteins were extracted, heat-inactivated and transferred onto PVDF membrane by electrophoresis. The membrane was blocked by 5% skimmed milk. After blocking and washing, the membrane was incubated with the primary antibody at 4°C overnight and then with the secondary antibody at room temperature for 2 h. Band intensity was measured by Tanon-5200 and Tanon MP software.

### IGF2BP3 siRNA

To silence IGF2BP3, three siRNAs (siIGF2BP3-1, siIGF2BP3-2, siIGF2BP3-3) were adopted in this study and were purchased from GenePharma, Shanghai. siIGF2BP3-1 has sense sequence of 5′-CCUUGAAAGUAGCCUAUAUTT-3′ and antisense sequence of 5′-AUAUAGGCUACUUUCAAGGTT-3′. siIGF2BP3-2 has sense sequence of 5′-GCAGGAAUUGACGCUGUAUTT-3′ and antisense sequence of 5′-AUACAGCGUCAAUUCCUGCTT-3′. siIGF2BP3-3 has sense sequence of 5′-GCUGGAGCUUCAAUUAAGATT-3′ and antisense sequence of 5′-UCUUAAUUGAAGCUCCAGCTT-3′. For non-targeting control, the Negative Control siRNA with sense sequence of 5′-UUCUCCGAACGUGUCACGUTT-3′ and antisense sequence of 5′-ACGUGACACGUUCGGAGAATT-3′ was used.

### Cell Proliferation Assay

ES2 or OVMANA cells were plated into three 96-well plates at 5 × 10^3^ cells/well and treated with NC, siIGF2BP3-1, or siIGF2BP3-2 using lipofectamine. Cell viability was determined at 24, 48, and 72 h, respectively, by using Cell Counting Kit-8 (CCK-8). Ten microliter of CCK-8 was added to each well and the plates were incubated for 1 h at 37°C. Finally, the three plates were inspected for absorbance at 492 nm.

### Crystal Violet Assay

Cells were digested and re-suspended in 1 mL binding buffer. Ninety microliter trypan blue and 10 μL cells were mixed and the cells were counted under a microscope. The cells were then seeded into a 24 well culture plate at a density of 4.5 × 10^4^ cells/well. After 24 h, binding buffer was replaced by 1 mL NC, siIGF2BP3-1, or siIGF2BP3-2 with lipofectamine in culture medium. After another 48 h, cells were fixed by 4% PFA and dyed by 1% crystal violet, and the plates were left at room temperature for 24 h for drying. Three hundred microliter 1% SDS was added to each well to exclude the dye and the OD values were measured for absorbance at 570 nm.

### Wound Healing Assay

Cells were seeded into a 24 well culture plate at 500 μL/well medium. After 24 h, the cell monolayer was washed by PBS and gapped with a pipette tip. The wound areas were marked for orientation and photographed as 0 h. Different treatments were added to each well. Marked wounds were photographed at 24 h and quantified. Five randomly selected points along each wound were marked, and the horizontal wound widths were measured using Primo Vert, Carl Zeiss, Germany. The migrated distance of cells was determined by measuring the wound width at 24 h and subtracting it from the wound width at 0 h.

### Nude Mice *in vivo* Experiments

BALB/c female nude mice, 16 ± 2 g and 6–8 weeks old, were purchased from VRL, Beijing, China. All animal experiments were executed in accordance with the Institutional Ethics Committee of Harbin Medical University. The rearing environment had purified laminar air at 25 ± 2°C. A total of 7 × 10^5^ ES2 cells in a volume of 80 μL cells, treated with NC or siIGF2BP3 by lipofectamine transfection, were injected into either of the hind legs. We monitored the mouse body weight every alternative day. When the xenograft lumps could be observed, the tumor volume was recorded by the same person with the same method. All the mice were sacrificed at the end of experiments (Day 25) according to ethics, and the data of tumor volume, tumor weight, and body and spleen weight were collected simultaneously for use in statistical analysis. The tumor tissues were fixed in 4% paraformaldehyde and subjected to pathologic and immunohistochemical examinations.

### Statistical Analysis

Associations of IFG2BP3 expression with clinicopathological parameters were examined using the *Chi square* test for categorical variables and analysis of variance for continuous data. Kaplan-Meier survival analysis was performed with endpoints of ovarian cancer specific and overall survival for AOVT and overall survival for the COEUR cohort. Cox proportional hazard regression model was used to estimate hazard ratios (HR) and 95% confidence intervals (CI) for the association between IGF2BP3 expression and overall survival adjusted for age, stage, and cohort. Molecular data were presented as the mean of triplicate or quadruplicate determinants with standard error (S. E.). JMP (SAS Statistical) version 13.0.0 was used for exploring clinical data and generating survival curves. Assays were repeated at least three times. Statistical analysis was performed to assess the differences between the means of the Negative control and siIGF2BP3 using Student's *t*-test, *Chi square* test, and Spearman's Rank correlation analysis with SPSS statistical software version 17.0 and GraphPad Prism software. *P*-value < 0.05 was considered statistically significant. Our study closely followed the guideline of randomness and preciseness to ensure reproducibility.

## Results

### IGF2BP3 Protein Expression in the AOVT and COEUR Cohorts

**Figure 1:**
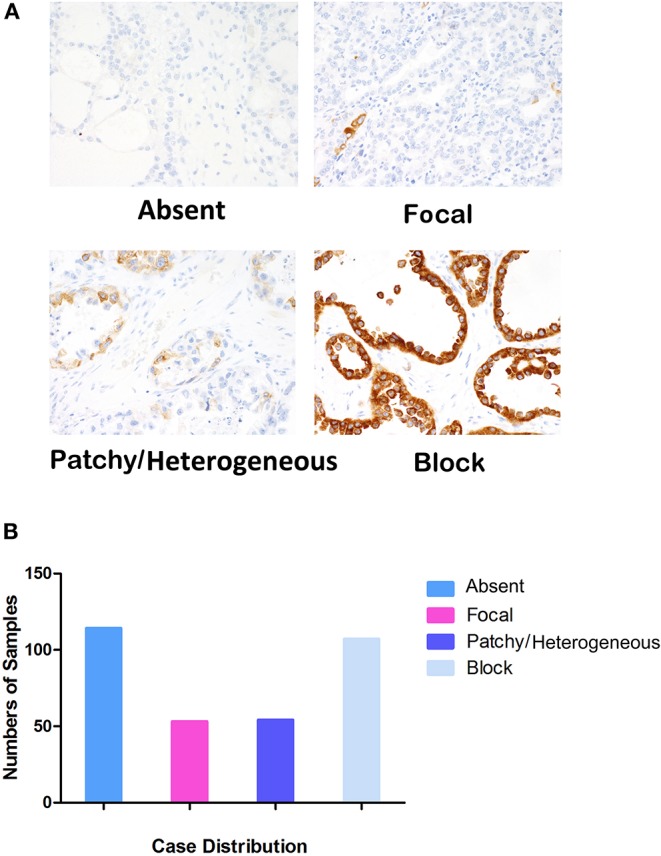
Evaluation standards of IGF2BP3 in IHC and case distribution. **(A)** Four levels of IGF2BP3 expression (×400). **(B)** Case distribution of different IGF2PB3 expression levels, Absent (*N* = 114), Focal (*N* = 53), Patchy/Heterogeneous (*N* = 54), and Block (*N* = 107).

**Figure 2:**
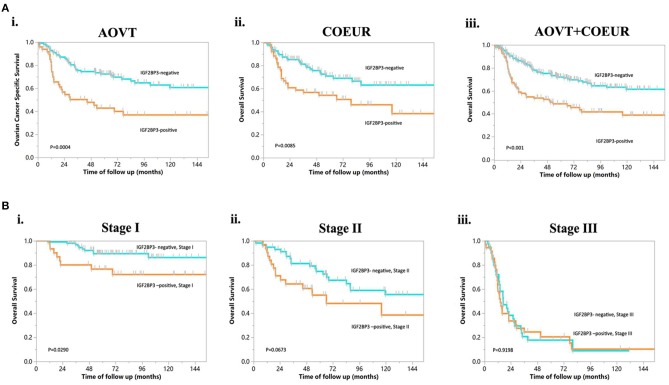
Kaplan-Meier curves of overall survival in OCCC. **(A)** IGF2BP3 expression in patients from different cohorts. (i) OCCC from AOVT, (ii) OCCC from COEUR, (iii) OCCC from both AOVT and COEUR. **(B)** IGF2BP3 expression in patients with different FIGO stages: (i) stage I, (ii) stage II, (iii) stage III.

Examples of staining and the distribution of IGF2BP3 expression for the 328 OCCC cases are shown in Figures 1A,B (Absent, *N* = 114; Focal, *N* = 53; Patchy/Heterogeneous, *N* = 54; Block, *N* = 107). Evaluation of the Kaplan-Meier curves using the 4-tier original interpretation of immunohistochemical staining suggested a categorization of the cases into two groups: cases with block staining (Positive, *N* = 107) vs. cases with non-block staining (Negative, *N* = 221, consisting of absent, focal, and patchy staining). There was near complete agreement (39/41, 95%) between the tissue microarray assessment and full sections regarding binary cut-off of positive/negative staining (i.e., the presence and absence of block staining). Two cases were interpreted as positive on full sections but negative on tissue microarray. According to the categorized IGF2BP3 expression data, the Kaplan-Meier survival curves demonstrate significant associations of IGF2BP3 overexpression with ovarian cancer specific survival in the AOVT and overall survival in the COEUR cohort (Figure 2A and Supplementary File 2.i). Because of the similar results, we combined both cohorts and performed a multivariate cox regression analysis: HR = 1.59, 95% CI 1.09–2.33; adjusted for age, cohort, and stage. Additionally, we performed stratified Kaplan-Meier survival analysis by stage (Figure 2B and Supplementary File 2.ii). IGF2BP3 expression was significantly associated with overall survival in patients diagnosed at stage I (5-year survival rate 89.3%, SE = 3.4% for non-block vs. 76.5%, SE = 2.8% for block staining, log rank *p* = 0.029; HR = 2.70, 95% CI 1.03–6.85). IGF2BP3 expression was closely associated with clinicopathological parameters, with positive IGF2BP3 expression being frequently present at stage III (*P* = 0.0056) but no associations with age or treatment (Table 1).

**Table 1:** Correlations of the IGF2BP3 protein expression levels with the clinicopathological features of ovarian clear cell carcinoma.

**Characteristic**	**Total**	**IGF2BP3 expression**		***P***
		**Positive**	**Negative**	
All cases (*N*, %)	328	107 (32.62)	221 (67.37)	
Age (years)				=0.1049, NS
<60	213 (64.94%)	76 (71.02%)	137 (61.99%)	
≥60	115 (35.06%)	31 (28.98%)	84 (38.00%)	
FIGO stage				=0.0056^*^
I	144 (43.9%)	33 (30.84%)	111 (50.23%)	
II	90 (27.44%)	33 (30.84%)	57 (25.79%)	
III	80 (24.39%)	36 (33.64%)	44 (19.91%)	
Unknown	14 (4.27%)	5 (4.67%)	9 (4.07%)	
Chemotherapy				=0.1785, NS
Yes	274 (83.54%)	94 (87.85%)	180 (81.45%)	
No	32 (9.76%)	6 (5.61%)	26 (11.76%)	
Unknown	22 (6.71%)	7 (6.54%)	15 (6.79%)	
Radiotherapy				=0.2243, NS
Yes	34 (10.37%)	14 (13.08%)	20 (9.05%)	
No	140 (42.68%)	39 (36.45%)	101 (45.70%)	
Unknown	154 (46.95%)	54 (50.47%)	100 (45.25%)	

* *P < 0.01*.

### Expression Levels of IGF2BP3 in OCCC Cell Lines and Normal Ovarian Cells

**Figure 3:**
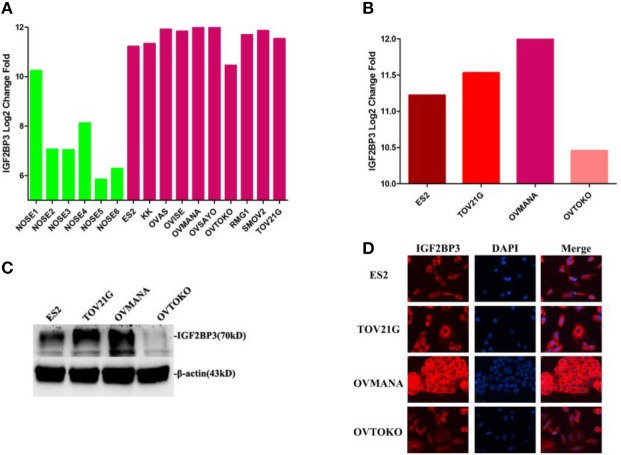
Expression levels of IGF2BP3 in OCCC cell lines and normal ovarian cells. **(A)** IGF2BP3 gene expression profiles of 6 independent NOSE cultures and 10 ovarian clear cell lines in GSE16570. **(B)** IGF2BP3 gene expression profiles in four specific OCCC cell lines. **(C;** Supplementary Figure 2**)** The protein expression levels of IGF2BP3 in four OCCC cell lines. **(D)** IGF2BP3 localizes in cytoplasm of OCCC cells by immunofluorescence.

To further understand the IGF2BP3 expression levels in various OCCC cell lines, we compared the IGF2BP3 gene expression profiles of six independent normal ovarian surface epithelium (NOSE) cultures and 10 OCCC cell lines in GSE16570 (https://www.ncbi.nlm.nih.gov/geo/query/acc.cgi?acc=GSE16570). We found that the expression of IGF2BP3 was increased significantly in OCCC cell lines compared to NOSEs by Log2 (change fold) values (Figure 3A). The aforementioned results agreed with our clinical findings that IGF2BP3 was highly expressed in cancer cells compared to normal cells.

We then selected four OCCC cell lines (ES2, TOV21G, OVMANA, OVTOKO) for further analysis. As shown in Figure 3B, IGF2BP3 mRNA was overexpressed in ES2, TOV21G, and OVMANA cells lines, whereas the expression was relatively weak in OVTOKO (Figures 3C,D). IGF2BP3 was localized in the cytoplasm of OCCC cells as revealed by immunofluorescence (Figure 3E). Consistently, the protein expression of IGF2BP3 in the four cell lines revealed by Western blot assays and immunofluorescence both showed trends similar to the bioinformatics results (Figures 3C–E). Therefore, we chose ES2 and OVMANA for IGF2BP3 knockdown investigations.

### Knockdown of IGF2BP3 by IGF2BP3 siRNA in ES2 and OVMANA Cell Lines

**Figure 4:**
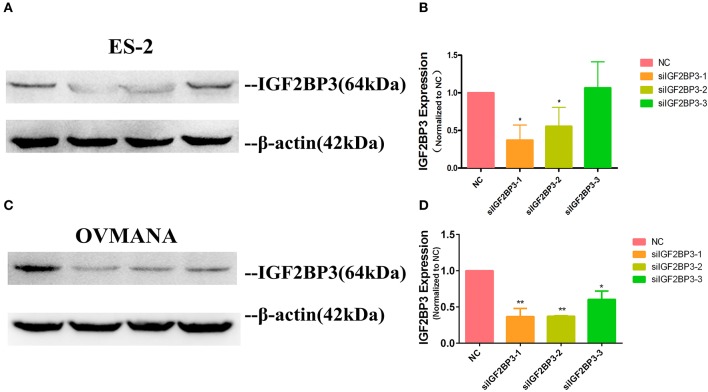
The expression level of IGF2BP3 after transfection with siRNAs. **(A;** Supplementary Figures 3A–C**; C;** Supplementary Figures 3D–F**)** IGF2BP3 protein expression after exposure to NC, siIGF2BP3-1/2/3 were verified by Western blotting in both ES2 and OVMANA cell lines. **(B,D)** Both siIGF2BP3-1 and -2 remarkably reduced IGF2BP3 expression, while -3 was not as significant as siIGF2BP3-1 and -2.*P < 0.05, **P < 0.01 compared with NC.

As high expression of IGF2BP3 was closely associated with poor survival in OCCC, we elucidated the effects of IGF2BP3 on OCCC cell proliferation, migration, and invasion. We conducted knockdown experiments and transfected ES2 and OVMANA or non-specific control cells with siRNA targeting IGF2BP3. We designed three siIGF2BP3s, including siIGF2BP3-1, siIGF2BP3-2, and siIGF2BP3-3, for this study. In ES2 cell line, the expression of IGF2BP3 was markedly reduced by siIGF2BP3-1 (37%) and siIGF2BP3-2 (55%) compared to NC (100%), but no significant difference was found after siIGF2BP3-3 treatment (Figures 4A,B). In OVMANA cell line, the expression of IGF2BP3 showed a trend similar to that of the ES2 cell line (Figures 4C,D). We thus used siIGF2BP3-1 and siIGF2BP3-2 for further validation.

### Effects of siIGF2BP3 on OCCC Cell Proliferation

**Figure 5:**
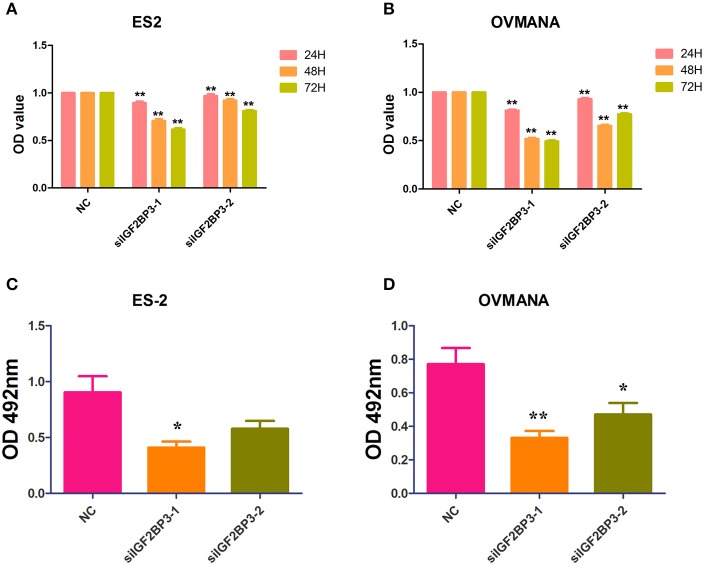
Growth inhibition of ES2 and OVMANA cell lines by siIGF2BP3 revealed by CCK8 and crystal violet assay. **(A)** Cell proliferation index determined after NC, siIGF2BP3-1, siIGF2BP3-2 for 24, 48, or 72 h in ES2 cells. **(B)** Cell proliferation index determined after NC, siIGF2BP3-1, siIGF2BP3-2 for 24, 48, or 72 h in OVMANA cells. OD450 value has been normalized to 1 in each negative control group. **(C)** OD570 value after different treatments in ES2 cells. **(D)** OD570 value after different treatments in OVMANA cells. **P < 0.01.

CCK-8 and crystal violet assays were performed to assess the effects of siIGF2BP3 on the proliferation potential of ES2 and OVMANA cells. As shown in Figure 5A, cell proliferation was significantly reduced by siIGF2BP3-1 and siIGF2BP3-2 at 24, 48, and 72 h in ES2 cells, and similar results were obtained with the OVMANA cell line (Figure 5B). According to the OD values, siIGF2BP3-1 (ES2: 0.8951, 0.7062, 0.6165; OVMANA: 0.8132, 0.5198, 0.4948) had stronger inhibitory effects than siIGF2BP3-2 (ES2: 0.9697, 0.9224, 0.8124; OVMANA: 0.9291, 0.6540, 0.7755) at 24, 48, and 72 h, respectively. Additionally, crystal violet assays showed that OD429 values were markedly decreased by siIGF2BP3-1 (ES2: 0.4094 ± 0.0535; OVMANA: 0.3310 ± 0.04087) and siIGF2BP3-2 (ES2: 0.5798 ± 0.06843; OVMANA: 0.4713 ± 0.0685) in both ES2 and OVMANA cell lines (Figures 5C,D). Crystal violet staining was decreased by siIGF2BP3 treatment compared with NC (Figure 5E).

### Effects of siIGF2BP3 on OCCC Cell Migration and Invasion

**Figure 6:**
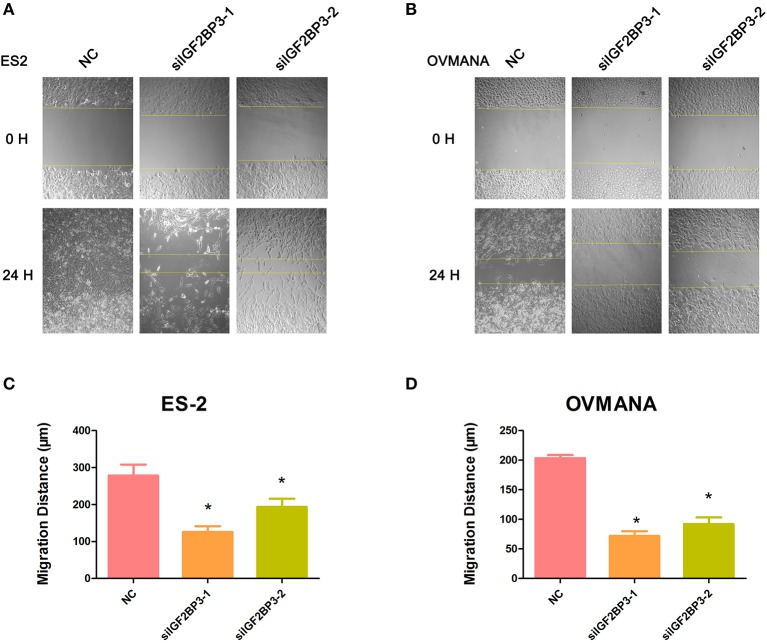
Effects of siIGF2BP3 on OCCC cell migration. **(A,B)** ES2 and OVMANA cells were scratched and treated with NC, siIGF2BP3-1, or siIGF2BP3-2. Photomicrographs were taken at 0 and 24 h after scratching. **(C,D)** Quantitation of wound healing assay by NC, siIGF2BP3-1, or siIGF2BP3-2, respectively. The migrated distance of cells was determined by measuring the wound width at 24 h and subtracting it from the wound width at 0 h. Results were obtained from three independent experiments, *P < 0.05, compared with NC.

**Figure 7:**
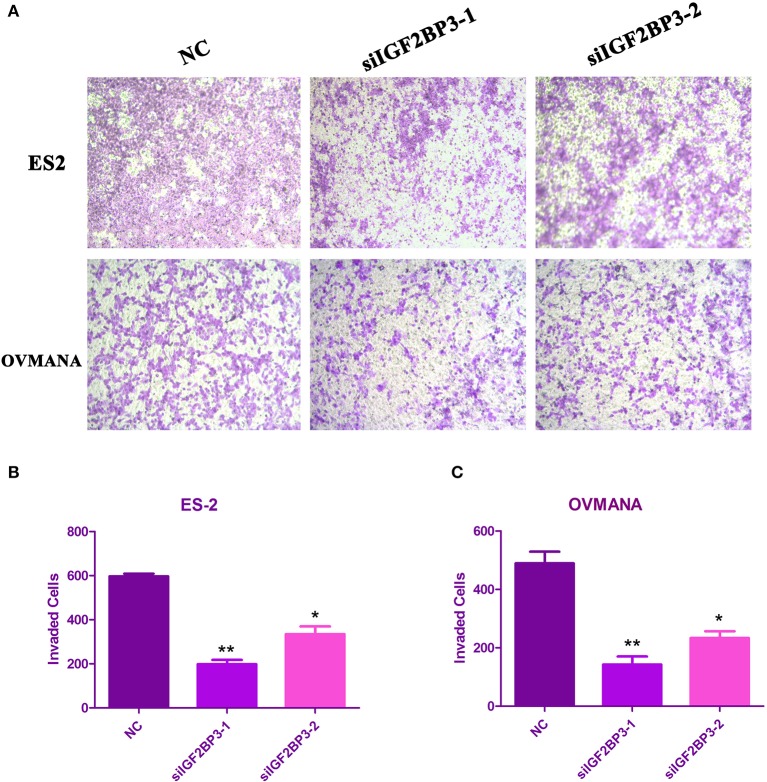
Effects of siIGF2BP3 on OCCC cell invasion. **(A)** ES2 and OVMANA cells were plated in the upper chamber with NC, siIGF2BP3-1, or siIGF2BP3-2. Pictures were taken under the microscope after 48 h of treatments. **(B,C)** Quantitation of invading cells. Results were obtained from three independent experiments, *P < 0.05, **P < 0.01, compared with NC.

We evaluated the effects of IGF2BP3 knockdown on cell migration by wound healing assays. As shown in Figures 6A,B, both ES2 and OVMANA cell lines treated with siIGF2BP3 showed decreased cell migration. The relative migration distances were measured after 24 h, and the migration ability was significantly weakened by treatment with siIGF2BP3 compared to negative controls in both ES2 and OVMANA cell lines (Figures 6C,D). As shown by the Transwell assays, siIGF2BP3-1/2 treatment greatly reduced the number of cells that had invaded the membrane, compared with negative controls (Figures 7A–C). These data together demonstrate that IGF2BP3 knockdown could inhibit ovarian cancer cell migration and invasion.

### Effects of siIGF2BP3 on OCCC Cell Apoptosis

**Figure 8:**
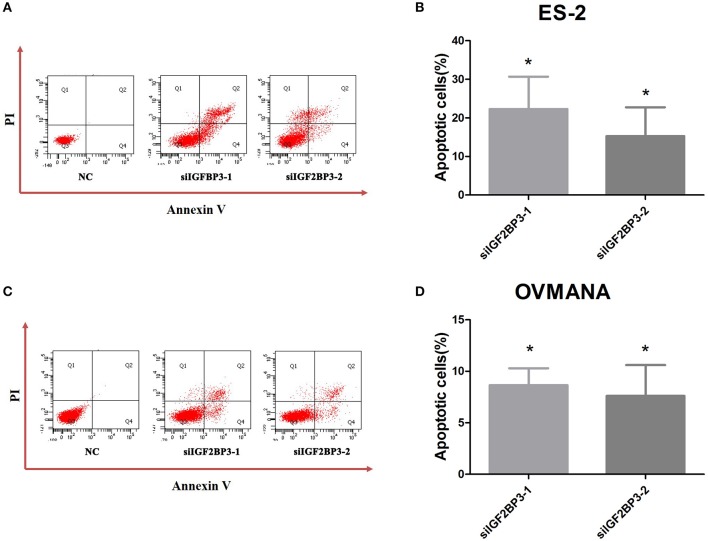
Effects of siIGF2BP3 on OCCC cell apoptosis. **(A,C)** Flow cytometry analysis after transfection with NC, siIGF2BP3-1, or siIGF2BP3-2 in both ES2 and OVMANA cell lines. **(B,D)** Quantitative analysis of apoptotic cells (%). Results were obtained from three independent experiments. NC group has been normalized as 0. *P < 0.05, compared with NC.

To determine the possible effects of IGF2BP3 knockdown on cancer cell apoptosis, we treated the OCCC cells with NC, siIGF2BP3-1, or siIGF2BP3-2 and detected apoptotic cells after siIGF2BP3 treatment in both ES2 (siIGF2BP3-1: 22.3%; siIGF2BP3-2: 15.23%) and OVMANA (siIGF2BP3-1: 8.65%; siIGF2BP3-2: 7.6%) cell lines (Figure 8). These data demonstrate that knockdown of IGF2BP3 could induce cell apoptosis significantly.

### The Expression Levels of MMP2 and MMP9 Were Markedly Down-Regulated by siIGF2BP3

**Figure 9:**
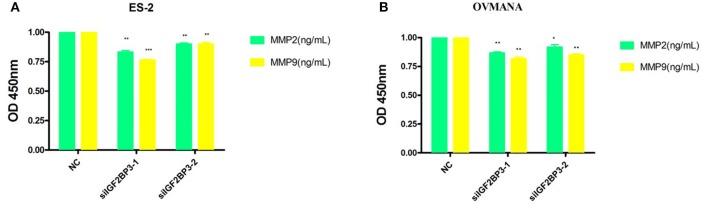
The effect of siIGF2BP3 on MMP2 and MMP9 levels in both ES2 and OVMANA cell supernatants. **(A)** Bar chart of ELISA analysis of MMP2 and MMP9 levels in ES2 cell supernatant. **(B)** Bar chart of ELISA analysis of MMP2 and MMP9 levels in OVMANA cell supernatant. Results were obtained from three independent experiments, *P < 0.05, ** *P* < 0.01 and *** *P* < 0.001, compared with NC.

**Figure 10:**
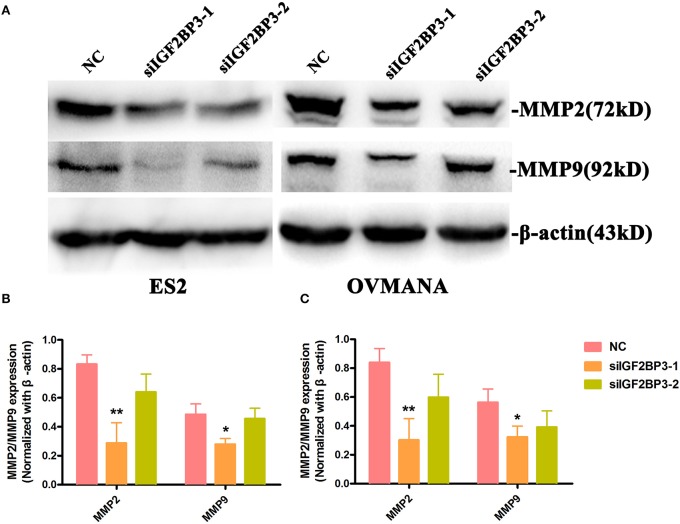
The effect of siIGF2BP3 on MMP2 and MMP9 protein expression in both ES2 and OVMANA cells. **(A)** Western blotting analysis of MMP2 and MMP9 protein in ES2 (Supplementary Figure 4) and OVMANA (Supplementary Figure 5) cells. **(B,C)** Relative expression levels of MMP2 and MMP9 protein in ES2 **(B)** and OVMANA **(C)** cells. Results were obtained from three independent experiments. MMP2 and MMP9 expression level comparison, *p < 0.05, **p < 0.01.

To further establish the functions of IGF2BP3 in OCCC, we used MMP2 and MMP9 as markers of metastasis. MMP2 (72 kDa type IV collagenase) is intimately linked with the invasion and metastasis of ovarian cancer, while MMP9 (68 kDa type IV collagenase) is a useful serum marker of ovarian cancer. MMP2 and MMP9 levels in culture media of the cell lines were determined by ELISA assays. Compared with the negative control (Figures 9A,B), siIGF2BP3-1 and -2 significantly down-regulated MMP2 and MMP9 expression levels in ES2 and OVMANA cells. We conducted Western Blot analysis to evaluate the expression intensity of the MMPs in the two cell lines after treatment with the products for 24 h and found that the expression levels of MMP2 and MMP9 were remarkably inhibited by siIGF2BP3 compared to NC (Figures 10A–C).

### IGF2BP3 siRNA Exerted Tumor-Suppressive Effects *in vivo*

**Figure 11:**
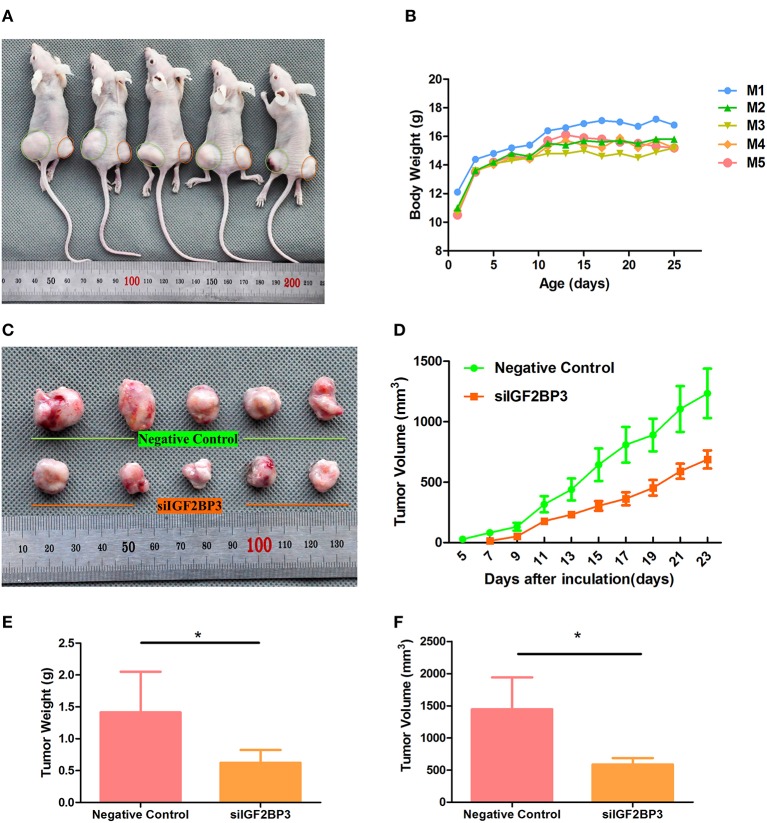
Tumor growth suppression *in vivo* by siIGF2BP3. **(A)** The subcutaneous xenograft tumor models were generated by transplanting human ovarian cancer ES-2 cells into nude mice. **(B)** Body weight changes over the 25 days of tumor growth. **(C)** Tumor growth at day 25 by NC and siIGF2BP3-1 treatments. **(D)** Tumor volume changes over the 25 days of tumor growth. **(E)** The removed tumors were weighed and statistically analyzed (*n* = 5). The data represent the means ± SD. *P < 0.05, vs. NC. **(F)** Tumor volume comparison among the animals of the two groups at day 25. The data represent the means ± SD. *P < 0.05, vs. NC.

As *in vitro* experiments showed that siIGF2BP3-1 had better inhibitory functions than siIGF2BP3-2, we used the former for *in vivo* analysis. ES2 cells transfected with negative control or siIGF2BP3 were subcutaneously injected into the hind legs of nude mice (Left: NC; Right: siIGF2BP3). Compared with that in the left side (negative control, Day 5), the tumor in the right side (treated with siIGF2BP3-1) delayed tumor growth by 2-days (Day 7) (*P* < 0.05, Figures 11A,C,D), while all animals gradually gained body weight (Figure 11B). At the end of the experiment, all mice were sacrificed for histological examinations of the tumor tissues. We found that siIGF2BP3 impeded tumor growth as reflected by both tumor weight and volume (*P* < 0.05, Figures 11E,F) with no effects on body weight. siIGF2BP3 showed a greater reduction of tumor weight and volume than negative controls, suggesting that inhibiting IGF2BP3 could delay the tumor growth *in vivo*.

### IGF2BP3 siRNA Suppressed Tumorigenesis by Down-Regulating MMPs and PCNA

**Figure 12:**
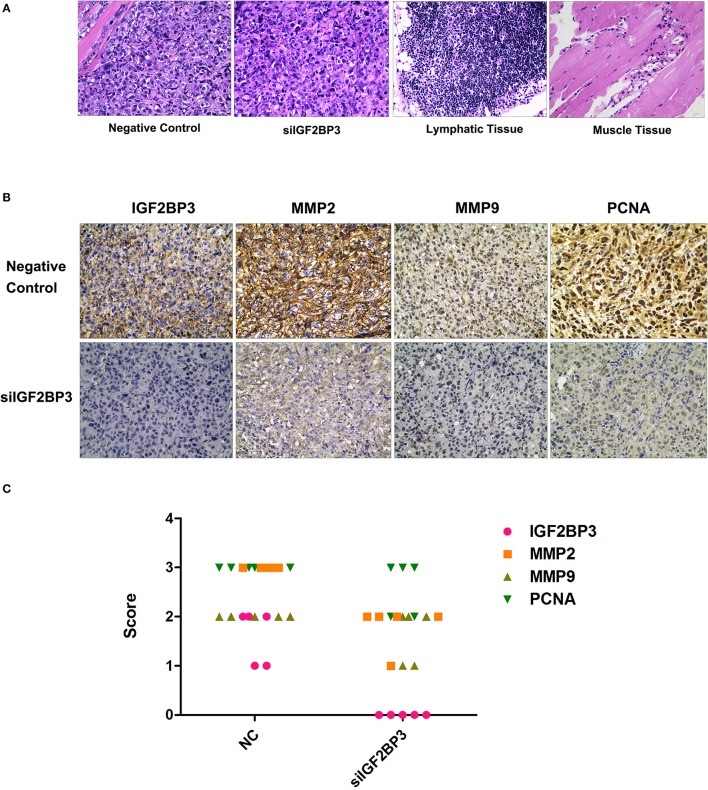
H&E and Immunohistochemistry staining for formalin-fixed paraffin-embedded tumor sections. **(A)** Neoplasm in negative controls has invaded and destroyed the basic structure of adjacent smooth muscle. There was no lymph node metastasis. **(B)** Expression levels of IGF2BP3, MMP2, MMP9, and PCNA in tissue blocks. Cancer cell cytoplasm staining was positive IGF2BP3, MMP2, and MMP9 were expressed in both membrane and cytoplasm while only nuclear staining was considered PCNA-positive. **(C)** Immunohistochemical scores were authorized by two pathologists under strictly double-blind method: the percentage and intensity of immunoreactive cancer cells <5% (score 0), 5~25% (score 1), 26~50% (score 2), 50~75% (score 3), and >75% (score 4).

The hematoxylin-eosin staining morphology of IGF2BP3 siRNA treated tumors was not different from the control (Figure 12A). In the siRNA group, MMP expression levels were relatively reduced compared to those of the NC group (Figures 12B,C). These results further confirmed that IGF2BP3 had an ability to accelerate tumor progression through up-regulating MMP expression, while siRNA could reverse it. Furthermore, the PCNA expression level also decreased in treatment group, suggesting siRNAIGF2BP3 could inhibit cell proliferation directly (Figures 12B,C).

## Discussion

In this study, we validated IGF2BP3 as a prognostic marker for OCCC using two large cohorts with 328 cases in total. A potential clinically useful finding is that IGF2BP3 overexpression stratified stage I OCCC patients regarding to their outcomes. The management of stage I OCCC represents a clinical challenge. These patients have an approximate 90% 5-year survival rate. Prognostic markers inferring the biology of the disease can inform the decision of whether to give adjuvant therapy. The 5-year survival rate of patients with stage I OCCC and IGF2BP3 overexpression is only 76%, indicating that these patients require adjuvant therapy. Since chemotherapy is ineffective (31), radiotherapy might be considered for treatment of localized disease in these patients (32). Hence, based on our results, further validation of IGF2BP3 for clinical risk stratification of OCCC at stage I is recommended before a potential clinical application. IGF2BP3 may be part of a multimarker panel (33, 34). Notably, IGF2BP3 overexpression was not associated with survival in stage III. Stage III OCCC patients showed a 2-year survival of <40%, reemphasizing the need for new therapeutic strategies for OCCC. However, IGF2BP3 overexpression was more common in stage III; together with the prognostic value seen in stage I and II, it indicates a more aggressive biology in OCCC. As an important refinement to prior studies, we changed the cut-off for “positive” IGF2BP2 expression from present to diffuse block staining. This was necessary because of the greatly increased sensitivity of new generation immunohistochemical platforms, such as the Dako Omnis, due to the introduction of polymer-based detection systems (35). Using this high cut-off, we showed near perfect concordance between the TMA interpretation and full section staining, the latter being clinical practice. This should allow for smooth clinical implementation.

Although this study offers confirmation of IGF2BP3 as a robust biomarker to predict the aggressive clinical behavior of OCCC, its cellular functions should be elucidated further. First, we observed that IGF2BP3 was highly expressed in OCCC cell lines compared to normal ovarian epithelial cells, a finding consistent with its role as oncofetal proteins, which are not expressed in normal adult tissue. We chose two IGF2BP3 high expressing OCCC cell lines, ES2 and OVMANA, for knockdown experiments by siIGF2BP3. Knockdown of IGF2BP3 inhibited cell proliferation, migration and invasion, and in the meantime enhanced apoptosis, which is in line with the ability of IGF2BP3 to serve as a post-transcriptional regulator on a variety of targets and cellular functions (10). We also demonstrated that IGF2BP3 inhibition led to reduced tumor growth *in vivo*. A limitation of the siRNA *in vivo* experiment is that the tumor cells were only briefly exposed to siRNA, which was then followed by *in vivo* growth for 23-days. The siRNA effect will diminish after a few days so that we believe that the reduced tumor size was due to a transient inhibition, which amounted to a delayed start in tumor growth. Further, we reported another mechanism by which IGF2BP3 knockdown reduced the MMP2 and MMP9 protein levels in OCCC, as shown in triple negative breast cancer before (20). Because IGF2BP3 is able to bind multiple target RNAs, thereby altering their stability, translatability and/or localization, we predict that it may function directly or indirectly to modify the expression of the various gene products, including MMPs, PCNA or other proteins involved in the control of cell proliferation, death, migration and invasion as shown in this study. Further studies will be needed to identify precisely the downstream effectors of IGF2BP3 in OCCC.

## Conclusion

In summary, IGF2BP3 overexpression in OCCC was associated with unfavorable overall survival independent of standard clinical parameters. IGF2BP3 as a prognostic biomarker should be further validated especially in patients diagnosed at Stage I. Furthermore, siRNA knockdown experiments using OCCC cell lines demonstrated a variety of *in vitro* and *in vivo* parameters indicating the down-regulation of MMPs, supporting a role of IGF2BP3 in tumor progression as a potential oncogenic agent. For future directions, we propose mRNA expression analyses to identify main downstream effectors for IGF2BP3 action in OCCC by comparing IGF2BP3 positive and negative OCCC.

## Data Availability Statement

All datasets generated for this study are included in the article/Supplementary Material.

## Ethics Statement

The studies involving human participants were reviewed and approved by Health Research Ethics Board of Alberta (HREBA)—Cancer Committee (CC). The patients/participants provided their written informed consent to participate in this study. The animal study was reviewed and approved by Health Research Ethics Board of Alberta (HREBA)—Cancer Committee (CC); Collage of Pharmacy, Harbin Medical University.

## Author Contributions

HL and MK designed the study and drafted the manuscript. ZZ, CL, MA, and SW controlled analyzed and interpreted data. MK participated in sample collection and scored the TMA sample. SL, LK, and HY carried out the immunohistochemical experiment. WX, QX, and WM did *in-vitro* and *in vivo* experimental process. ZZ, PW, JW, and EM contributed reagents, materials, and analysis tools. RJ contributed reagents, materials, analysis tools, and supervised HL as a co-supervisor at the University of Calgary. RJ, S-LL, and MK revised this article. S-LL finalized the manuscript. All authors read and approved the final manuscript.

### Conflict of Interest

The authors declare that the research was conducted in the absence of any commercial or financial relationships that could be construed as a potential conflict of interest.

## Supplementary Material

Supplementary Figure 1 Immunohistochemical stains for IGF2BP3 in TMA. **(A)** IGF2BP3 expressed in different tissues as strong positive (a placenta tissue)/weak positive (a germinal center tissue)/negative (a normal liver tissue) controls (×100). **(B)** IGF2BP3 expressed in CCC (×100), lower left- IGF2BP3 negative expression, lower right- IGF2BP3 positive expression (×400).

Supplementary Figure 2 The expression level of IGF2BP3 in different OCCC cell lines.

Supplementary Figure 3 The independent triplicate results of IGF2BP3 in ES2 and OVMANA cell lines after transfection with siIGF2BP3-1/2/3.

Supplementary Figure 4 The independent parallel results of MMP2 and MMP9 in ES-2 cell line.

Supplementary Figure 5 The independent parallel results of MMP2 and MMP9 in OVMANA cell line.

Supplementary File 1 Clinicopathological features information.

Supplementary File 2 Risk number distributions.

## Abbreviations

OCCC: Ovarian clear cell carcinoma
IGF2: Insulin-like growth factor-II
IGF2BP3/IMP3: Insulin-like growth factor-II mRNA-binding protein 3
COEUR: Canadian Ovarian Experimental Unified Resource
AOVT: Alberta Ovarian Tumor Type
TMA: Tissue microarrays
CCK8: Cell counting kit 8
ELISA: Enzyme-linked immunosorbent assays
FCM: Flow cytometry
MMP2: Matrix metalloprotein 2
MMP9: Matrix metalloprotein 9
NOSEs: normal ovarian surface epithelial cell preparations
NC: negative controls
HR: hazard ratios
CI: confidence intervals
S. E.: standard error.
