# Supplementary figures and images for: Overexpression of IGF2BP3 as a Potential Oncogene in Ovarian Clear Cell Carcinoma
Source: Front Oncol. 2020 Jan 30;9:1570. doi: 10.3389/fonc.2019.01570 (PMC7002550; doi:10.3389/fonc.2019.01570)

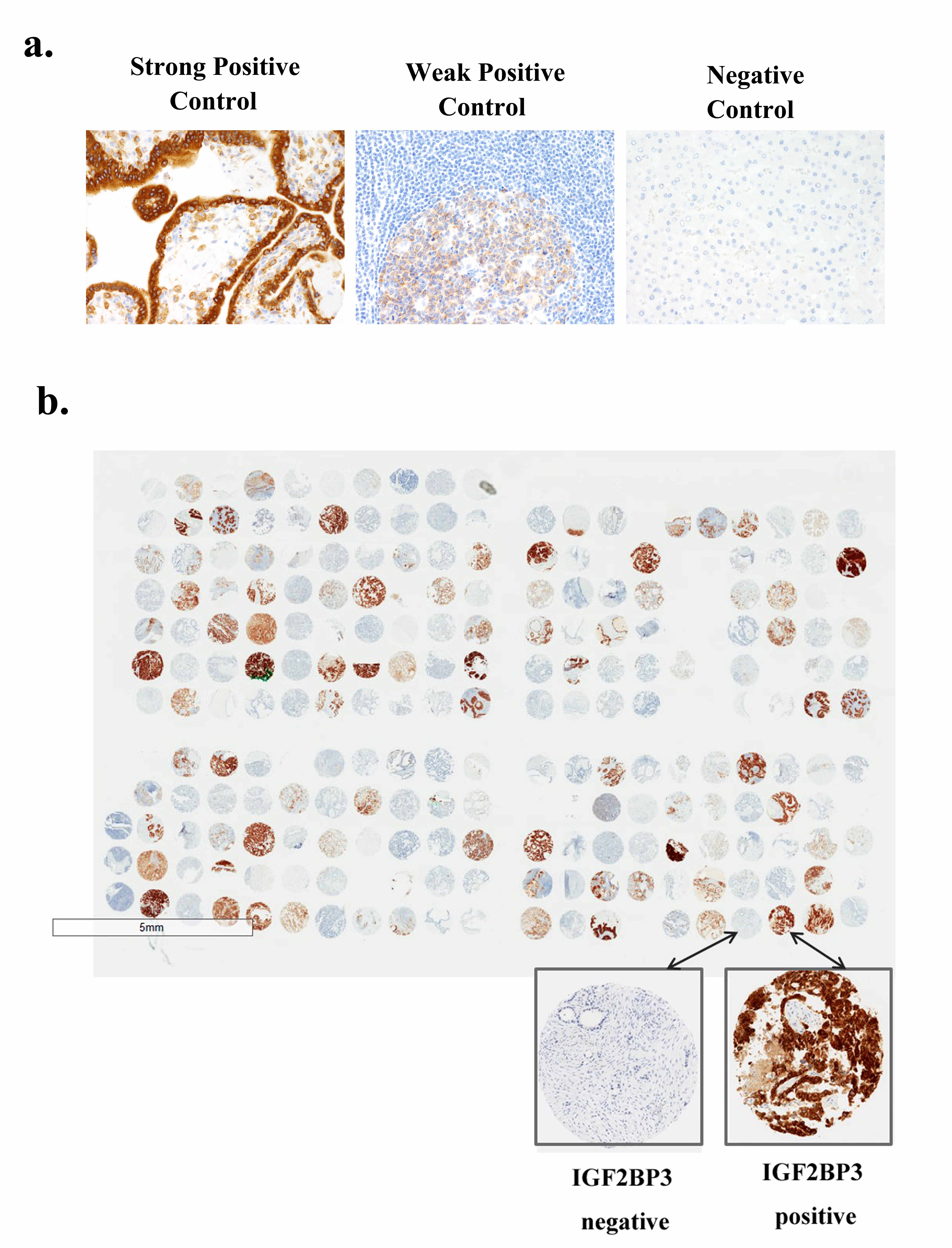

Supplement: Supplementary Figure 1 — Immunohistochemical stains for IGF2BP3 in TMA. (A) IGF2BP3 expressed in different tissues as strong positive (a placenta tissue)/weak positive (a germinal center tissue)/negative (a normal liver tissue) controls (×100). (B) IGF2BP3 expressed in CCC (×100), lower left- IGF2BP3 negative expression, lower right- IGF2BP3 positive expression (×400). [file Image_1.JPEG]

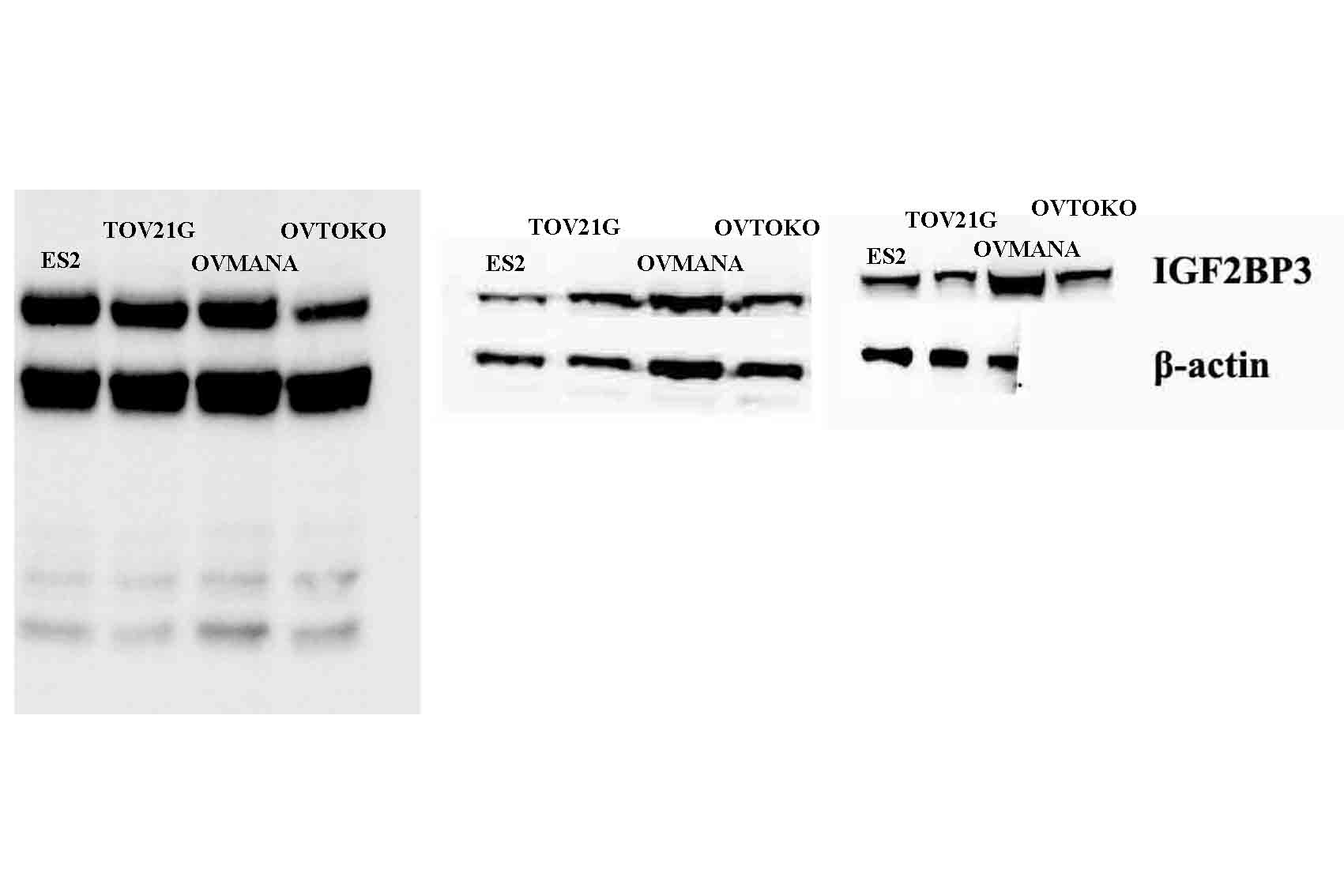

Supplement: Supplementary Figure 2 — The expression level of IGF2BP3 in different OCCC cell lines. [file Image_2.jpeg]

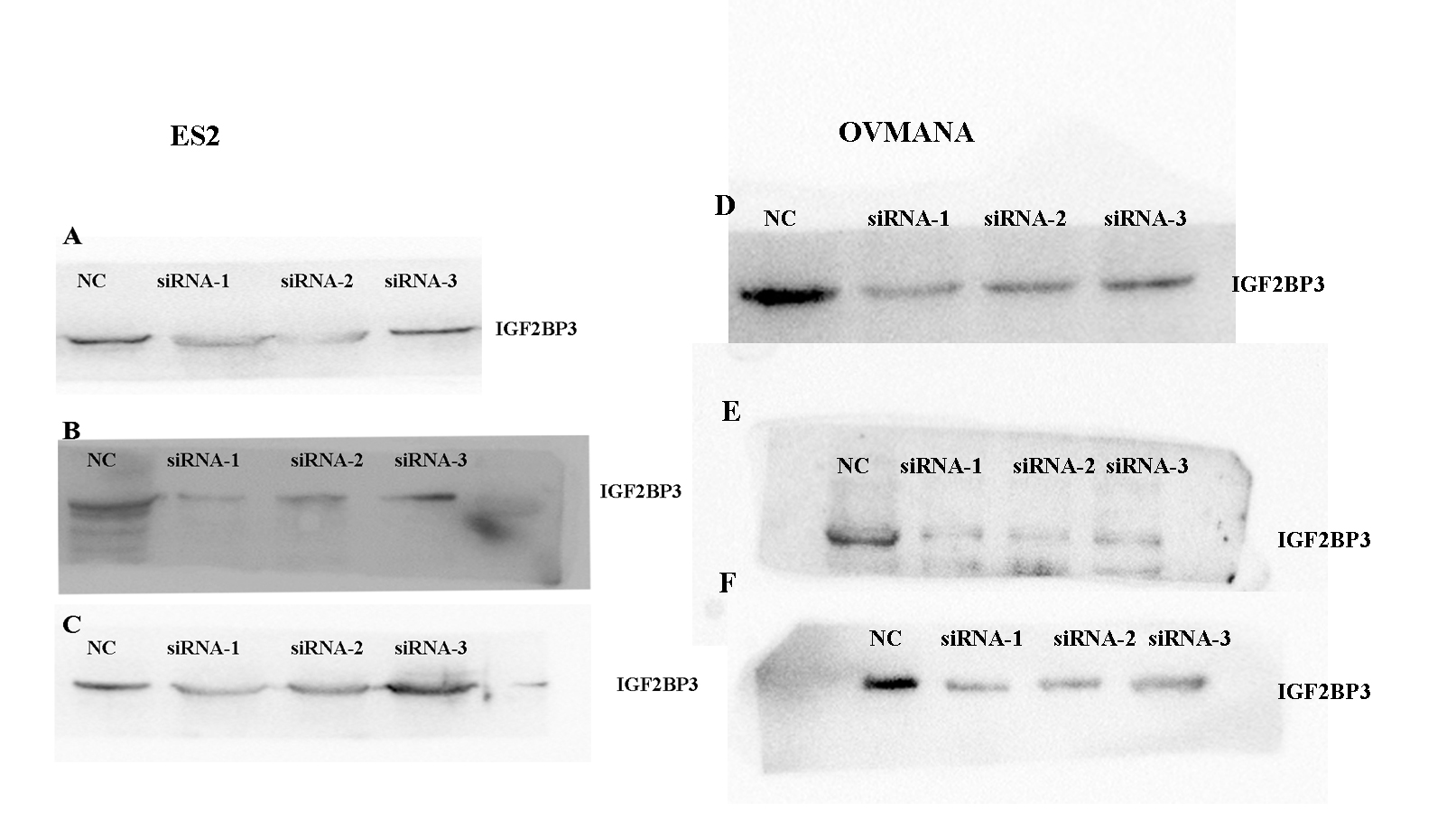

Supplement: Supplementary Figure 3 — The independent triplicate results of IGF2BP3 in ES2 and OVMANA cell lines after transfection with siIGF2BP3-1/2/3. [file Image_3.jpg]

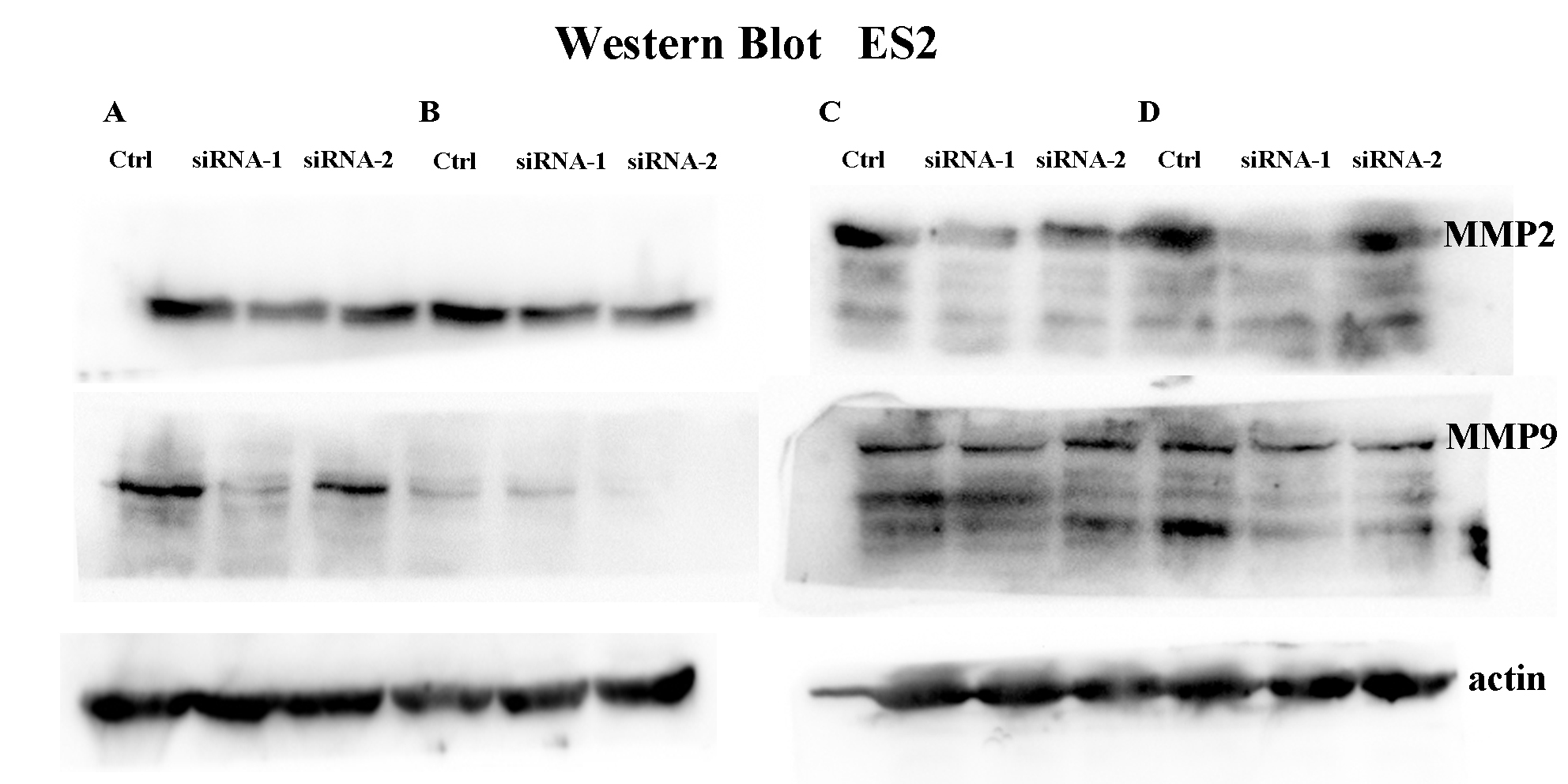

Supplement: Supplementary Figure 4 — The independent parallel results of MMP2 and MMP9 in ES-2 cell line. [file Image_4.jpeg]

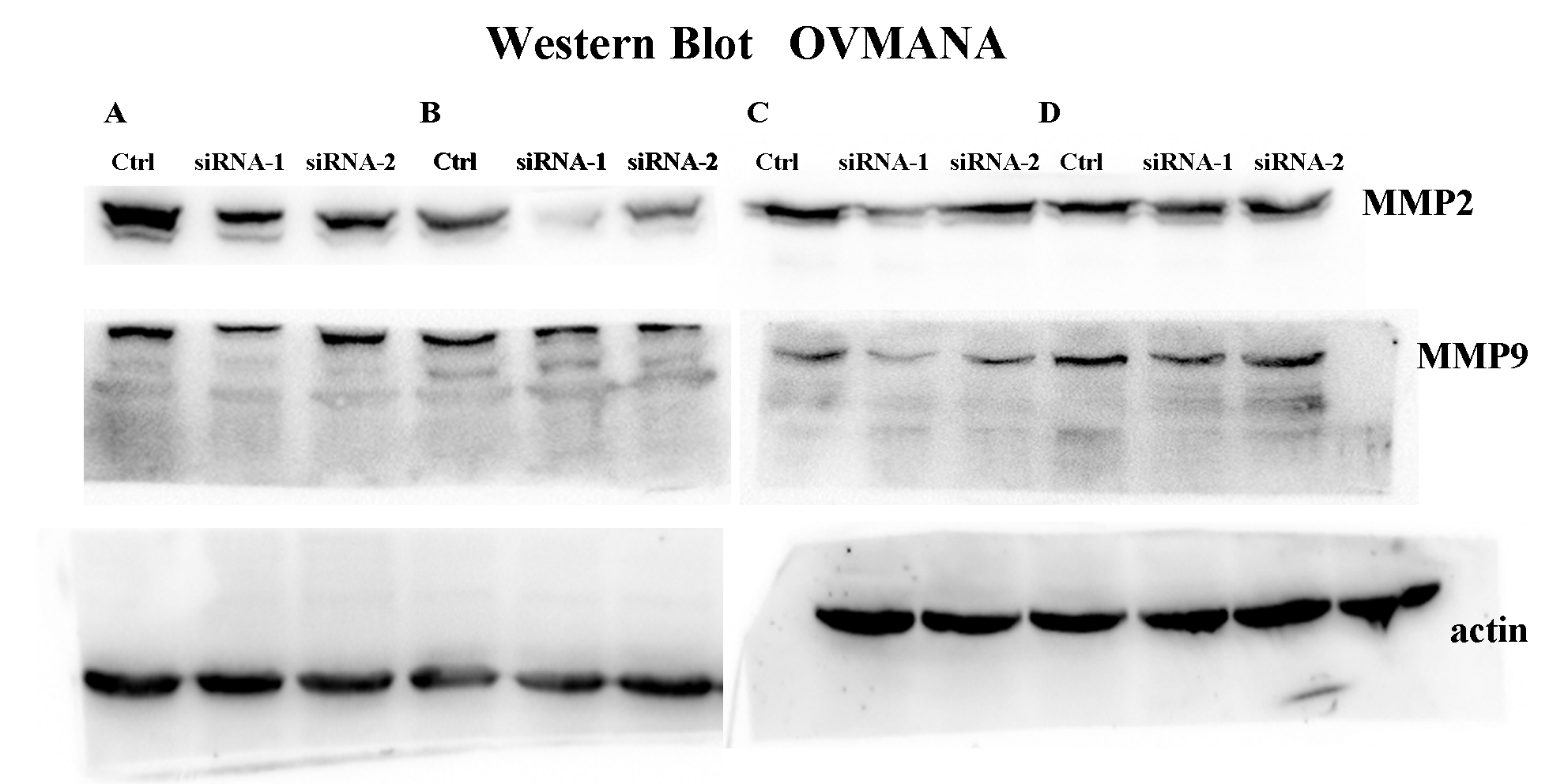

Supplement: Supplementary Figure 5 — The independent parallel results of MMP2 and MMP9 in OVMANA cell line. [file Image_5.jpeg]
