# Supplementary material for: Overexpression of IGF2BP3 as a Potential Oncogene in Ovarian Clear Cell Carcinoma
Source: Front Oncol. 2020 Jan 30;9:1570. doi: 10.3389/fonc.2019.01570 (PMC7002550; doi:10.3389/fonc.2019.01570)
Supplement: Supplementary File 2 — Risk number distributions. [file Table_2.DOCX]

**Supplementary file 2. Risk Number Distributions**

**i.**

| Cohort | IGF2BP3 | Case  No. | No. at Risk (Month/Cases) | | | | | | |
| --- | --- | --- | --- | --- | --- | --- | --- | --- | --- |
|  |  |  | **0** | **24** | **48** | **72** | **96** | **120** | **144** |
| AOVT | **Negative** | 113 | 113 | 92 | 75 | 49 | 36 | 29 | 21 |
|  | **Positive** | 49 | 44 | 27 | 21 | 15 | 11 | 10 | 7 |
| COEUR | **Negative** | 108 | 108 | 76 | 54 | 33 | 20 | 8 | 5 |
|  | **Positive** | 58 | 57 | 32 | 25 | 13 | 8 | 6 | 2 |
| AOVT+COEUR | **Negative** | 221 | 140 | 89 | 71 | 45 | 33 | 28 | 20 |
|  | **Positive** | 107 | 73 | 24 | 18 | 14 | 11 | 10 | 7 |

**ii.**

| Stage | IGF2BP3 | Case No. | No. at Risk (Month/Cases) | | | | | | |
| --- | --- | --- | --- | --- | --- | --- | --- | --- | --- |
|  |  |  | **0** | **24** | **48** | **72** | **96** | **120** | **144** |
| I | **Negative** | 111 | 111 | 96 | 76 | 46 | 31 | 17 | 13 |
|  | **Positive** | 33 | 33 | 25 | 24 | 16 | 10 | 9 | 4 |
| II | **Negative** | 57 | 57 | 51 | 41 | 27 | 20 | 17 | 12 |
|  | **Positive** | 33 | 33 | 23 | 15 | 8 | 7 | 5 | 4 |
| III | **Negative** | 44 | 44 | 14 | 6 | 3 | 2 | 2 | 1 |
|  | **Positive** | 36 | 36 | 12 | 8 | 5 | 3 | 3 | 2 |
| Unknown | **Negative** | 9 | 9 | 9 | 8 | 7 | 5 | 3 | 2 |
|  | **Positive** | 5 | 4 | 1 | 1 | 1 | 1 | 1 | 1 |
